# ‘High schools High on life’: Development of an Intervention to Reduce Excessive Drinking in Danish High Schools

**DOI:** 10.3389/fpubh.2020.00435

**Published:** 2020-09-15

**Authors:** Veronica Sofie Clara Pisinger, Sofie Have Hoffmann, Lotte Pålsson, Peter Dalum, Morten Klöcker Grønbæk, Janne Schurmann Tolstrup, Lau Caspar Thygesen, Rikke Fredenslund Krølner

**Affiliations:** ^1^National Institute of Public Health, University of Southern Denmark, Copenhagen, Denmark; ^2^The Danish Cancer Society, Copenhagen, Denmark

**Keywords:** alcohol, school, intervention, adolescents, social norms, parents, school environment

## Abstract

**Background:** Clear documentation of the understanding of the problem, process of development, and content of interventions is essential to enable other researchers to understand why interventions succeed or fail and to enable researcher to build on previous evidence and replicate and adapt findings in other contexts. In this paper we describe the rationale, intervention development, and final design of the ‘High schools High on life’ intervention; a high school-based, multi-component intervention to reduce excessive drinking among Danish high school students.

**Methods:** The development of the intervention ‘High schools High on life’ was guided by the planning steps of the Intervention Mapping protocol (IM) in combination with the behavior change wheel and the behavior change techniques, theory, evidence, practice, and new empirical studies of contextual factors in the Danish high school setting.

**Results:** The development process resulted in a multi-component intervention with the following intervention elements: a school environmental component targeting school alcohol policies and norms, a school educational component addressing students' social norms around alcohol, and a parental component encouraging parent-child communication around alcohol.

**Discussion:** Not all steps of IM were followed rigidly. However, IM proved useful as a planning tool in combination with the behavior change wheel and the behavior change techniques, as it provided a systematic approach to the intervention development process. IM forced the research group to be explicit about decisions and choices throughout the planning process. The transparency of the developmental process and theoretical, empirical and practical/contextual foundation of the ‘High schools High on life’ intervention may enable future intervention studies to build on our findings and accumulate knowledge to reduce excessive drinking among young people.

**Trial registration:** The trial was registered at clinicaltrials.gov (Trial registration number NCT03906500) prior to randomization.

## Background

Denmark has one of the highest prevalence of drunkenness among adolescents in Europe (1). Among Danish high school students (15–20-year olds) 28 % have reported binge drinking [drinking five or more units (one Danish unit of alcohol is defined as 12 grams of pure alcohol) at one occasion] four or more times within the last 30 days prior to answering the survey, and 20 % indicated drinking above the Danish Board of Health's high risk drinking limits for adults (21 units a week for men and 14 units a week for women) (2). In the short-term, alcohol use in adolescence can lead to injuries, homicide, suicide, violence, criminal activity, poor health, and risky sexual behavior (3). In the long-term excessive alcohol use in adolescence often tracks into and through adulthood increasing the risk of alcohol related morbidity, mortality and risk of high alcohol consumption and alcohol dependence later in life (4–8).

The school has been proposed to be one of the most effective settings to reach a substantial number of young people at risk of developing harmful drinking levels. Numerous school-based substance abuse prevention programs have been developed and tested and some have been effective in postponing debut age or reducing consumption (9–11). Interventions targeting older adolescents (15–20-year old) are mostly American college interventions (12, 13), high risk interventions based on screening and brief motivational interviewing (14, 15) or web-based personalized normative feedback interventions (16, 17). However, evidence from the American college literature is difficult to transfer to the Danish high school setting as alcohol is a strongly integrated part of the school culture, and a large group of Danish students drink excessively with the purpose of getting drunk (18, 19). Danish students, of all ages, are allowed to drink and buy alcohol at high school parties (18). Previous Danish interventions have been targeting younger age groups and have not been effective in reducing excessive drinking (20, 21). Thus, there is a need for developing new interventions targeting excessive drinking in the Danish high school context with easy implementation into existing organization and culture.

School-based interventions about alcohol are often complex and multifaceted which challenge the identification of active, effective components within them (9, 22). A comprehensive description of the development of the intervention and the programme theory is important to understand the mechanisms by which interventions are expected to lead to behavior change (23). A detailed and transparent description of the decision process will demystify the logic between program objectives, intervention strategies and their theoretical underpinnings and gain insight into the working mechanisms of the actual program (22). The ‘High schools High on life’ intervention was developed as a multi-component high school-based intervention with the aim of reducing excessive drinking among Danish high school students. The aim of this paper is to describe the development and design of the intervention.

## Methods

### Intervention Development

The ‘High schools High on life’ intervention was developed in collaboration between the Center for Intervention Research (CIR) at the National Institute of Public Health, University of Southern Denmark and the Danish Cancer Society (DCS). The DCS was responsible for the development of the practical intervention components and materials. The CIR was responsible for securing a theoretical foundation and evidence base of the intervention design, including needs assessment, identification of the most important and modifiable determinants, feasibility testing of intervention ideas and outline of the program theory. Further, the CIR was responsible for the design and conduct of the effect- and process evaluation of the intervention (which is elaborated in the study protocol) (24).

The ‘High schools High on life’ intervention builds on a socio-ecological framework which recognizes that adolescents' drinking behavior is determined by a wide range of interacting factors on multiple ecological levels (25). Ecological models emphasize the structural, physical and political context while incorporating social and psychological influences. The intervention was therefore designed as a multilevel intervention, using multiple strategies to reduce excessive drinking among high school students. We combined school and parental components to ensure that students received consistent and coherent healthy messages in both settings. The development of the intervention consisted of six steps which were guided and inspired by the main principles from the Intervention Mapping (IM) protocol (26), although the protocol was not followed rigidly.

### Step 1. Logic Model of the Problem

Based on the previously described high prevalence of excessive drinking among Danish high school students (2) our intervention focused on prevention of excessive drinking. We began the IM process by stating the program goal i.e., defining that we wanted to develop an intervention to reduce excessive drinking among high school students. Based on effect estimates from the Unplugged program (27) a previous successful school-based substance abuse prevention program tested among junior high school students (12–14 year-olds) in seven European countries, we specified the program goal: At the end of intervention, the mean number of binge drinking episodes (*drinking 5 or more units of alcohol within one occasion)* within the last 30 days prior to answering the survey will be 30% lower among students who have received the ‘High school High on life’ intervention compared to students at schools without the intervention (control group).

Through reviews of both quantitative and qualitative research focused on risk factors for young peoples' drinking, we established a logic model of the problem (26, 28). We elaborate the main determinants for excessive alcohol consumption among adolescents by the ecological levels described below.

#### Individual Level

##### Knowledge

- Lacking knowledge of the school alcohol policy may affect drinking habits. Among Danish high school students 64% are aware that their school has an alcohol policy, though 97% of the school leaders stated that they have this type of policy (29).

##### Motives and outcome expectancies

- Danish adolescents drink to lose control, let their guards down, have fun, connect with friends, potential new friends, and enhance/initiate romantic relations (30–32).
- Drinking among adolescents may be seen as a “Rite de Passage” - of the transformation from childhood to adulthood (33) and a way to signal maturity (30, 33).
- Drinking may be used to cope with hard times, e.g., 39% of the Danish high school students agreed or totally agreed with the statement “*I drink to forget my problems”* (32).
- High school students are more concerned about short-term than long-term health consequences of drinking; many fear losing control, crossing personal boundaries, or doing something they would regret (31).
- The brain is under comprehensive development in late adolescence, which can lead to unrealistically low expectations of own risk and increasing risk-taking, especially in the presence of peers (34).

##### Personality factors

- Impulsive risk-seeking personality and externalizing problems have been associated with substance abuse in adolescence (35–37).

##### Sociodemographic

- Boys and older adolescents generally have higher alcohol consumption than girls and younger adolescent (32).
- Alcohol consumption increases with parental educational level and income in Denmark (32), while lower family socioeconomic position is associated with more alcohol-related harm (38).

#### Interpersonal Level

##### Peers

- Misperceptions that peers have higher rates of drunkenness and more positive attitudes toward alcohol consumption is a driving factor for young peoples' excessive drinking (30, 39).
- Some students drink to avoid social exclusion e.g., among the 15–25 year-olds 56 % think it is hard to be a part of the social community if they do not drink alcohol (40) and Danish high school students report using “cheating” strategies to avoid drinking, such as pretending they drink more alcohol than they actually do (31).

##### Parents

- Children of parents who have clear attitudes to alcohol consumption, and who communicate to their children that they do not think they should drink heavily, drink less in adolescence than children of parents with no clear attitude (41, 42).
- Children and young people drink less if their parents set limits of how much children are allowed to drink (41, 43).
- A close and supportive relationship with parents is associated with lower alcohol consumption in adolescents (41).
- Parents serve as role models for their children, and children are thus likely to copy their parents' alcohol consumption habits. Parents' alcohol use is therefore a determinant for their children's alcohol use (41, 42, 44).

#### School Level

##### The social context of alcohol intake in danish high schools

- Alcohol constitutes an assimilated element in many social activities constituting the core of Danish high school culture, such as high school parties and excursions (19).
- Heavily drinking at high school parties is encouraged by the organizing student party committee e.g., in written invitations to the parties as well as specific events/actions taking place during the school parties, as well as advertisements from local bars and clubs (45).

#### Community Level

##### Liberal policy and culture

- Higher consumption of alcohol among adults at the community-level has been associated with a higher prevalence of adolescent drunkenness in the community (46).
- Adolescents in communities that have easy access to alcohol, for example those with many outlets close to schools and with low or no control with legal purchasing age, are more likely to drink excessively (47, 48).

#### National Level

##### Liberal policy and culture

- In general Denmark has a liberal alcohol culture, a high national alcohol intake among adults, and one of the worlds' lowest proportion of abstainers (49).
- There is strong evidence that availability, price, and legal purchasing age are all important policy measures of adolescent alcohol use (49). In Denmark prices for alcohol are low and alcohol is sold 24/7 with a high density of outlets. Further, at age 16 you can legally buy alcohol with an alcohol percentage lower than 16.5% in outlets, whereas 18-year-olds can buy alcohol above 16.5% and be served alcohol in bars, restaurants etc.
- Alcohol marketing has also been linked to excessive drinking in youth (50–52).

### Context and Capacity Assessment

To elaborate the needs assessment and gain further insights into the role of alcohol in the high school setting, we conducted interviews with high school principals and high school students

We interviewed three school principals, about their experience with students drinking on high school grounds, alcohol policies, school-based initiatives to reduce drinking, and potential barriers for implementing these initiatives. Schools were selected based on their prior engagement and experience with alcohol prevention.

The interviews made us aware that an increasing number of schools are collaborating on mutual alcohol policies to reduce student's drinking. The policies are designed to limit availability of alcohol at the included schools by e.g., prohibiting alcohol with a high alcohol percentage (stronger than 5% pure alcohol) to be sold at regular school parties and denying entrance of drunk students at school parties. The collaboration on mutual alcohol policies was established to prevent distorted competition among schools on attracting future students based on alcohol polices. School principals were afraid that adoption of more restrictive alcohol policies than neighboring schools would reduce the number of future students applying for admission due to students' expectation of boring non-alcoholic parties. This potential barrier was further investigated in step 2.

Some of the interviewed school principals had experimented with alcohol free events, but student attendance had generally been low for these events. All interviewed school principals tried to collaborate with parents by informing parents about the school alcohol policy and encouraging parents to take responsibility for pre-parties. School principals generally received support from parents but found it difficult to balance involvement of parents with their values and primary educational task of cultivating students to be knowledgeable and responsible adults.

We conducted five focus group interviews (*n* = 19, 12 girls and seven boys, six first year-, seven second year- and six third year students) at four high schools about students' experience with alcohol consumption at school and their motives for drinking. Students were selected to represent diversity in perspectives, based on school year, sex, and alcohol consumption (also including non-drinking students). In line with the review of the literature, students reported drinking alcohol to have fun and to socialize with friends and engage in new relations (31). Students were more concerned about short-term consequences of alcohol intake such as passing out or getting sick and thereby missing out on the fun, than potential long-term health effects (31). Drinking to intoxication was generally accepted and friends helped each other if one had had too much alcohol. Students were concerned about social exclusion of non-drinking peers and peer pressure to drink. The students primarily got drunk at pre-parties prior to school events, but also consumed alcohol at the high school at events where alcohol was sold.

Members of the student party committees and the introduction committee were identified as important role models and influencers on social norms of alcohol use. Further, these students have a great influence on events and parties held at schools and whether alcohol plays a dominant role at these occasions. Members of the student party committees and the introduction committee were therefore selected as an intervention delivery group.

Selection of target group for the intervention: First year students (~16 years-old) were selected as the primary target group of the intervention, as alcohol consumption often escalates in the transition from primary school to high school (53, 54). In the beginning of high school 1st year students (15–16 years) meet new people, join new peer groups, and attend social events at and outside school. These experiences contribute to the formation of perceived norms about high school alcohol consumption. Further, we expected 1st year students to be less affected by prior alcohol policies and traditions at school, than 2nd and 3rd year students.

## Step 2. Program Outcomes and Objectives – Logic Model of Change

### Selection of the Most Important and Modifiable Determinants

We identified the most important and modifiable determinants and developed preliminary intervention components targeting these taking theoretical behavior change techniques into account. In the following section we summarize the selected determinants, performance objectives and outcomes, and describe the tests of preliminary intervention component ideas.

Based on the literature review and interviews with school principals and students and the resulting logic model of excessive drinking, we selected the most important and modifiable determinants of Danish high school students' drinking behavior. This resulted in a focus on the school environment including policies and norms, students' social norms and parents' knowledge and attitudes. Despite being important determinants, community and societal factors such as availability of alcohol and prices for alcohol outside the high schools were not targeted in the intervention, as we did not have the power to modify these factors. For each determinant, performance objectives and behavioral and environmental outcomes were specified (Table 1). Based on the behavioral and environmental outcomes, and behavior change theory, intervention ideas were suggested. For some performance objectives, the DCS had already developed materials that targeted determinants to meet these outcomes. The DCS adapted some of these materials to fit the high school setting, whereas others had already been tested feasible at high schools, though the intervention effects were unknown. Based on feedback from school principals on limited resources among teachers to facilitate the intervention, the intervention was designed to be largely implemented by school management, students and external resources instead of teachers.

**Table 1:** Determinant, Performance objectives and Behavioral and Environmental outcomes for Danish high school students' alcohol use.

**Determinants for alcohol use**	**Performance objectives**	**Behavioral and environmental outcomes**
**Individual and peer level**		
Knowledge	Information about the school alcohol policies	Students are aware of the school alcohol policy and short-term consequences (social and health related) of excessive alcohol consumption.
Motives	Create opportunity to socialize without drinking.	Student committees organize events and parties with less focus on drinking.
Outcome expectancies	Make it cool to drink less and uncool to be very drunk Make students reflect about their alcohol use and when it is fun and not fun to drink.	Students have less positive outcome expectancies of excessive drinking. Students engage in promoting the ideal of drinking less.
Social norms	Create awareness of alcohol consumption, peer pressure, and attitudes toward alcohol, among peers. Correct misperceptions of peers' attitude and behavior (reduce peer pressure on drinking)	Students are aware of the actual drinking level at their high school. Students experience to be able to have fun without drinking. Students are aware that most students accept and respect non-drinking or less drinking.
**School level**		
Alcohol is an integrated part of the high school culture.	Create and communicate clear alcohol policies to students and parents. Create better opportunities for socialization without alcohol. Educate student committees on their responsibility for the social environment	The high school has a clear alcohol policy and secures that students, parents and all staff are aware of the policy, and secures that the policy is enforced. The high school makes sure that student committees organize events and parties with less focus on drinking, and that 1st years students do not experience peer pressure on alcohol when starting high school. High school introduce a treatment referral strategy for students with potential problematic alcohol or other substance use.
Availability of alcohol	Limit the number of events where alcohol may be consumed. Limit alcohol with high alcohol percentage at school events.	High schools arrange no more than 5 parties and 1 dinner where alcohol may be consumed. High schools never serve alcohol with a percentage above 5% of pure alcohol.
Alcohol marketing	Control that heavy drinking is not encouraged within the high school.	High schools monitor that local pubs/bar/clubs do not advertise within school property and that excessive drinking is not encouraged in the promotion for events at the school.
**Parental level**		
Parental attitude to child's alcohol use	Make parents aware of their influence on and responsibility for their children's drinking.	Parents are aware of their responsibility and influence on their child's drinking.
Dialog on alcohol consumption	Encourage and provide parents with skills to discuss alcohol with their children and promote responsible drinking. Encourage high schools to inform parents about the school alcohol policy.	Parents talk to their child about alcohol, are aware of the high school's alcohol policy and promote responsible drinking.
Agreements on child's alcohol consumption and positive monitoring	Inspire parents to make agreement with children on alcohol consumption.	Parents and child make agreements on the child's alcohol use.

### Testing Ideas and Assumptions and Understanding Possible Implementation Barriers

To test preliminary intervention ideas and understand possible barriers for implementation, we interviewed school principals from five different high schools. We used the Danish National Youth study 2014 (55) to select potential early adopters (*n* = 2), schools that had positive experience with initiatives to reduce alcohol use, as well as potential slow adopters (*n* = 3), schools with particularly high alcohol intake. We included both city and rural high schools in different regions of the country to test geographical variations in barriers.

School principals were generally positive toward the intervention ideas and agreed that it was a worthy aim. As potential barriers school principals identified resource shortages as they were under pressure from a new educational reform and budget cuts. Principals were reluctant to spend too much time and resources on implementing the intervention as they did not perceive health promotion as their primary task. School principals therefore demanded a minimum of school hours allocated to the intervention by students and teachers and that the intervention could fit into existing organizational structure. Further, many organizations were interested in partnering with the high schools about different (preventive) projects, which were time consuming and not always perceived as high schools' a primary mission. Principals were positive about the idea of a strict alcohol policy, though they expected resistance from students and teachers. They sought evidence and arguments to rise the case against potential opponents. The communication to schools about the project therefore highlighted that the goals and methods of the project supported the schools' goals and practices, such as increasing students' well-being, community building, creating a good learning environment, and exhibiting social responsibility. As mentioned in step 1, some schools shared policies with neighboring high schools and were reluctant to change policy if the neighboring school did not, due to competition distortion. We tested this potential barrier by contacting principals from nine different high schools located in both urban and rural neighborhoods. These interviews showed that this was only a concern among some high school principals; however most high school principals were willing to change the alcohol policy independent of neighbor high schools if they found it appropriate.

## Results

### Step 3-4. Program Design and Production: The ‘High School High on life’ Intervention Programme

**Figure 1:**
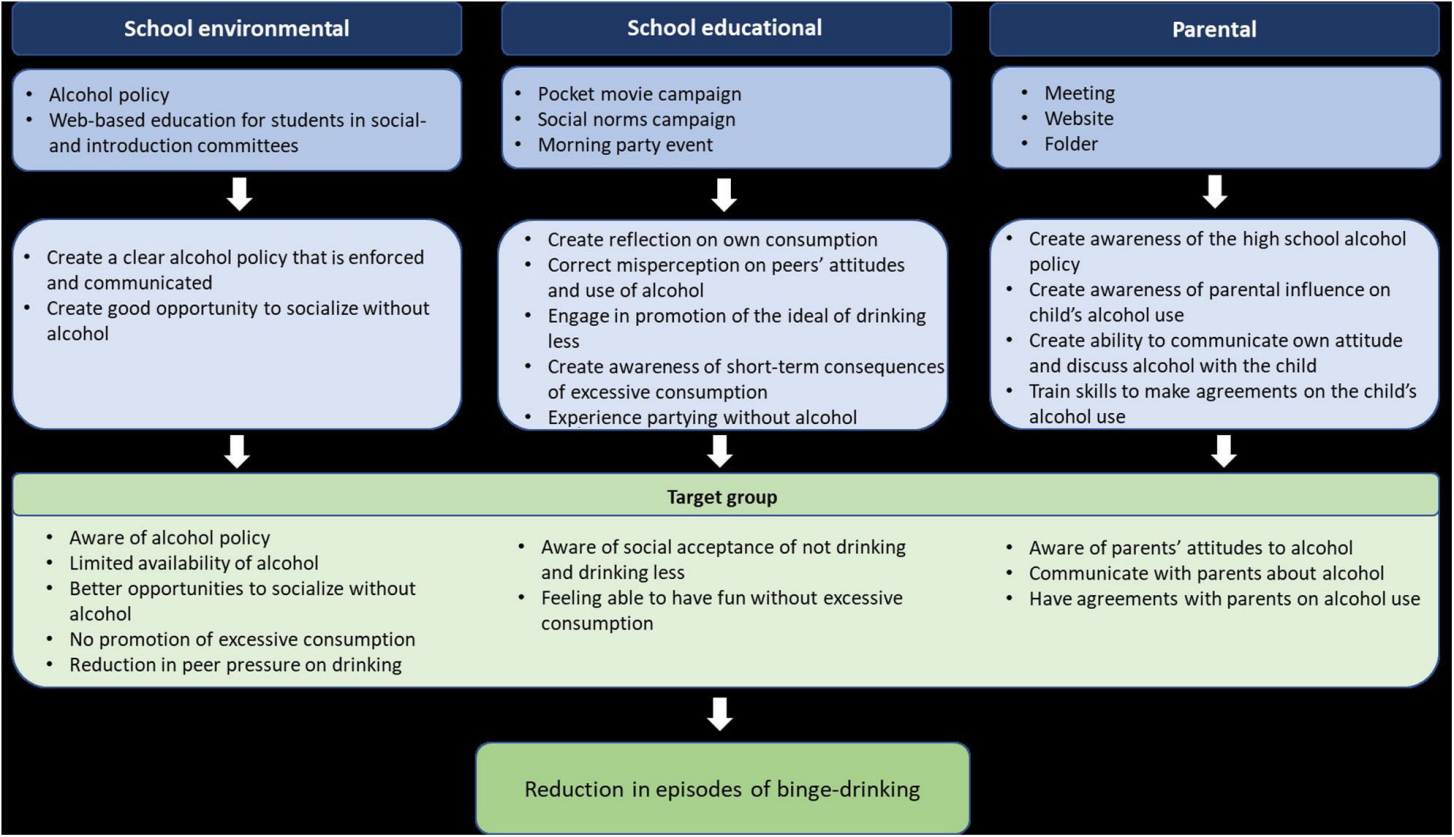
Programme Theory of the ‘High Schools High on life’ programme.

Based on the needs assessment, behavior change techniques (theoretical methods for change) and feedback from school principals and students, we settled on the final intervention program and components (Figure 1). The program theory builds on the COM-B model (56). The components were designed to give students new knowledge that would enhance their capability to change their alcohol use, to create new opportunities for behavior change by limiting availability of alcohol in the school environment and to create motivation to drink less by changing social norms. The DCS was responsible for the production of all printed and web-based materials. The DCS already runs a successful health promotion campaign called ‘*High on life*’ targeting Danish adolescents and young adults' (15–25-year-olds) alcohol use. The materials developed for the high school intervention build on the visual identity and creative universe of the campaign but were adapted to the high school setting. Project managers from the DCS ensured that the intervention material incorporated a pedagogical approach tailored to the target group (school principals, students and parents). All intervention materials were developed in collaboration with the research team at The CIR to ensure that environmental and behavioral outcomes were met.

An overview of the intervention components can be found in the Template of Intervention Description and Replication (57) in Table A1. Here we provide a detailed description of the intervention components, intervention functions (the “broad categories of means by which an intervention can change behavior” (22: p. 109) e.g., an intervention component may perform both persuasive and educational functions), policy categories which support the delivery of the intervention functions, behavior change techniques (the active ingredients which will bring about change), the target group, rationale, materials, mode of delivery, location, duration, and possible tailoring. Behavior change techniques were coded based on the Behavior Change Technique Taxonomy (22). Behavior change techniques were coded separately by two researchers (VP and RFK) and cases of discrepancies were discussed until unanimity was attained. Discrepancies were few, and mainly occurred as a result of different levels of insight into intervention components and unclear definitions of Behavior Change Techniques (BCTs). In the following section we will provide a short description of the school environmental, school educational, and parental components of the intervention.

### School Environmental Component

The school-environmental intervention component consisted of environmental and educational strategies designed to restructure the physical and social school environment which primarily served the intervention functions restriction, education and persuasion (22).

#### Alcohol Policy

A checklist for high schools' development of new alcohol policies (or revision of existing policies) was developed to limit students' access to alcohol at school, help enforcement, and communicate clear attitudes about alcohol to the students, parents, and teachers. The checklist was based on evidence from national alcohol policies, which have shown effect previously (58, 59). Further, to ensure feasibility the checklist development was guided by the previously mentioned mutual alcohol policies that exist at some collaborating high schools. These policies were further adapted after interviews with school principals. The final checklist is presented in Box 1. Most initiatives are mandatory to implement. A few initiatives are marked as optional because of expected implementation challenges or weaker documentation of effect. A local project coordinator at each school, was asked to revise the school alcohol policy in cooperation with school principals, based upon the checklist and to indicate the initiatives they intend to introduce, before school start August 2019. The project coordinators were asked to sign and return the adapted checklist to the research group and will be responsible for implementation and enforcement of the new policy.

Box 1 **Alcohol policy checklist.**

**Limited availability (mandatory unless stated otherwise)**

- Students are not allowed to drink or be affected by alcohol during school hours
- No alcohol is allowed at introductory events
- Introduction trips must be non-alcoholic
- Alcohol sold at social school events may maximum contain 5% pure alcohol
- Alcohol must be sold at maximum five yearly regular school parties and at the final graduation dinner
- Study trips must be non-alcoholic (optional)
- Alcohol must not be sold to students younger than 16 years at the school (optional)

**Security (mandatory)**

- The high school management has developed a strategy for referral to treatment of students they worry may have a substance abuse
- Responsible school staff is always present at social school events where alcohol is sold
- Professional guards are always present at school parties where alcohol is sold
- Students are denied entrance to school parties or sent home if they are visibly drunk. The school can test the blood alcohol concentration and send students home if its above 0.5

**Alternatives (optional)**

- Easy access to free water at events
- Access to alcohol-free alternatives (preferably drinks that look similar to alcoholic beverages sold, e.g. non-alcoholic beer)
- More non-alcoholic school events than events where alcohol is sold
- Café areas at parties where students can enjoy themselves with other activities than drinking alcohol such as playing games, etc.
- Ensuring sale of snacks/food at school events where alcohol is sold

**Communication (mandatory)**

- The school alcohol policy is communicated to parents, teachers, and students
- The school alcohol policy is available on the school website

#### Web-based Education for Students in Party- and Introduction Committees

To provide high school students with better opportunities for socializing without alcohol, two web-based education programs for the party and introduction committees were developed. Both programs were designed to provide skills, inspiration, guidance, and motivation to student committees to organize events and parties with less focus on drinking, and emphasize that 1st years students should not experience peer pressure to drink alcohol when starting high school. The programs were also designed to prompt the committee members to identify as role models for 1st year students. Systematic reviews and meta-analyses have shown that peer-led interventions are effective in preventing alcohol use among young people (60–62). Ideas and comments derived from five focus group interviews with student committee members guided the design of the introduction committee educational program and the social committee educational program. The education programs consisted of a dilemma-quiz where knowledge of Danish alcohol culture, peer pressure to drink, and relevant dilemmas are introduced as well as knowledge of rules and laws related to alcohol consumption in school settings. The web-based educational program for the social committee also included an online facilitated innovation process to guide the student party committee members to develop fun events/parties where alcohol is not the focus. Students from one party committee tested the online innovation workshop. The workshop was perceived acceptable and feasible.

The rest of the web-based educational materials was tested and perceived acceptable and feasible in two students' party committees and two introduction committees. Materials were adapted based on the students' feedback.

### School Educational Component

By use of the intervention functions: persuasion and education, the school-educational intervention component consisted of three elements designed to make 1st year students reflect on their alcohol use, correct misperceptions of peers' attitudes and behaviors in relation to alcohol use, increase students' awareness of the short-term consequences of alcohol (social and health related), decrease peer pressure to drink, and engage students in promotion of the ideal of drinking less.

#### Pocket Movie Workshop

The DCS had already developed and implemented a pocket movie workshop to high schools. The pocket movie workshop was a 1-day workshop where students make a short campaign movie using their smartphones. The workshop was developed to make students reflect about their alcohol use, and when it is fun and not fun to drink. The preliminary evaluation showed positive results in relation to students' attitudes about alcohol and this campaign was included as an intervention component. The pocket movie campaign was designed to change students' attitudes about alcohol by increasing their awareness of the short-term consequences of alcohol (social and health related) and engage them in promotion of the ideal of drinking less. Induced compliance approach theory (63) suggests that making adolescents arguing for a specific attitude such as “Drink less- experience more” can be effective in changing their attitudes and possibly also their behavior if it does not already comply with the induced attitude. Compared to a comparison group, Natvig and Aarø 2014 found a significantly lower increase in alcohol use among 8th grade students in Norway who competed class wise in producing the best 3-min alcohol prevention video aimed at convincing 7th graders not to start drinking (64). Consistent with the induced compliance approach to attitude change, involvement in producing the video influenced the 8th graders' own attitudes and drinking behavior.

All schools were offered a 1-day workshop for all 1st year high school students facilitated by the company Lommefilm A/S. In the workshop Lommefilm A/S demonstrated how to make movies using their smartphones and inform the students that consumption of alcohol increases the risk of short-term consequences such as conflicts, having sex you regret and injuries. In groups of four, students were encouraged to create a 45 s long prevention campaign movie with the message “Drink less- experience more.” To give students an incentive to participate, the best movie at each school was selected to participate in a national school competition. The winning high school received an alcohol-free party organized by the DCS.

#### Social Norms Campaign

The development of campaign materials was guided by the Social Norms Approach (65–67). Adolescents and young adults tend to overestimate drug use in their respective peer group and these incorrect perceptions are predictive of higher rates of personal drug use (68–70). These misperceptions may concern both rates of peer alcohol use (descriptive norms) and the social acceptability of alcohol use (injunctive norms). Previous interventions based on the social norms approach have been effective in changing attitudes and knowledge about the norms of alcohol use, and some have been effective in changing behavior (65, 69). Posters (digital and in print) were distributed to high schools to correct misperceptions about the group norm and thereby decrease the social pressure to drink excessively on the individual student. The ‘High school High on life’ posters included descriptive and injunctive norms tailored to each high school, based on the baseline survey. In a campaign video 3rd year high school students provided new 1st year students with advice about starting high school and encourage them to take it slow and not drink too much. Project managers were asked to share the video on the high school's websites, Facebook-pages, show it to new 1st year high school students in the beginning of the school year. Further, the video was available at the ‘High school High on life’ website. Through identification with 3rd year students, 1st year students are expected to change their expectation of how much they need to drink to fit in.

#### Morning Party Event

The DCS had already developed a morning party event for high schools. The objective of the morning party is to give students an experience of parting without drinking that can be transferred into practice at other parties. Interviews with students in step 1 showed lack of enthusiasm for hosting the event and students expressed practical challenges in terms of transport to schools with busses not running early in the morning. Therefore, the morning party event was voluntary for implementation in the schools. The project coordinator was responsible for hosting the event and was offered supported by the DCS in terms of help with planning, organizing and a budget on 10,000 Danish Krones (corresponding to ~1,300 EUROs or 1450 USD). Further, the morning party event could be held at the time of day that suits the high schools the best.

### Parental Component

Based on evidence of parents' influence on adolescents drinking (41–43) the parental component was designed to make parents aware of their influence on and responsibility for their children's drinking, encourage and enable them to discuss alcohol with their children, and to make them aware of the school's alcohol policy. The DCS had developed information materials for parents of primary school children prior to our study. This material was expanded and adapted for parents of high school students based on interviews with parents (*n* = 3).

#### Information Meeting

In the beginning of the school year, schools were encouraged to invite all parents to 1st year high school students to a parent meeting in order to introduce them to the school alcohol policy. At the meeting the parents were encouraged to support the school policy and discuss alcohol use with their children.

#### Information Folder

At the school meeting the parents should receive an information folder about high school students' alcohol use and attitudes, and what they, as parents, can do to prevent heavy drinking among their children. The information folder also included a link to the information website (described below).

#### Information Website

The website included a video of high school students discussing how they like their parents to make rules about their alcohol use and support them. Additionally, videos of psychologists explaining how to discuss alcohol with your child, and games with facts about adolescents' alcohol use are included.

### Step 5. Program Implementation Plan

Based on knowledge gained from the previous steps (capacity assessment and test of intervention ideas and assumptions), we developed a plan for implementation of the intervention and specified tasks and responsibilities of school staff and student committees as well as strategies to facilitate implementation of each intervention component (26). Each high school appointed a local project coordinator to be responsible for intervention implementation and maintain contact to the research team. To help ensure implementation each school principal should also appoint a local school coordinator for the social committee and the introduction committee from school staff (school administration or teachers). The students committee coordinator's tasks were to ensure that the student committees complete the web-based education programs and help the student introduction and party committee arrange events (introduction program for new 1st year students/cafés and parties) with no encouragement or pressure to drink. The components were designed to be easily implemented in school settings with minimal resources needed from the school administration and teachers. Prior to the implementation of the intervention, researchers visited all intervention schools to introduce the study rationale, the multiple intervention components, tasks, and evaluation framework to ensure the school principals and local coordinators understood all intervention tasks. All intervention materials was handed out in print, on a USB-stick for each school, and made available through dropbox and the project website: www.cancer.dk/gymnasierfuldafliv. To support the use of materials, the DCS developed implementation guides for the online education programs and the alcohol policy checklist. To create commitment, provide guidance and ownership throughout the intervention period, schools received newsletters with stories from intervention schools about local initiatives and press stories about the project from the media. Researchers monitored and supported the implementation at each school through frequent telephone calls, visits, newsletters, and e-mail reminders to local coordinators.

### Step 6. Evaluation Plan

Prior to initiation of the intervention development process, it was decided that we would test the effectiveness of the resulting high school-based intervention in a cluster-randomized controlled trial to obtain the highest level of evidence of the effect of the intervention (71). The use of a control group increase the chance that any changes observed at follow-up can be attributed to the intervention instead of other influences on students' binge drinking behavior in the intervention period. The cluster-design was chosen as we will intervene in the school environment and to reduce the risk of contamination between school classes we randomized entire high schools. Parallel to the process of intervention development (steps 1–5), we developed detailed plans for effect and process evaluation of the study including specification of main evaluation questions, concepts and measures (e.g., primary outcomes, background factors, prognostic variables, potential effect modifiers, determinants (potential mediators), unintended side effects, and process measures) the most suitable data collection methods and tools to inform the specific concept/measure (quantitative, qualitative or mixed methods approach) and the most reliable data source for the specific topic of evaluation (students, school principals, own observations) taking into account the burden on respondent, timing of data collection and analysis plans. The randomized trial was registered prospectively at clinicaltrials.org (Trial registration number NCT03906500). The effectiveness of the intervention and implementation strategies will be evaluated simultaneously (72). We will monitor how the intervention components are implemented to be able to explain the working mechanisms of the intervention and the effect or lack of effect. The development of the process evaluation study will be guided by a 6-step protocol for systematic process evaluation developed by Aarestrup et al. (73) and Grant et al.'s (74) framework for process evaluation of cluster randomized trials of complex interventions. We will combine qualitative and quantitative methods to evaluate different aspects of the process of implementation. Further information about the evaluation plan, may be found in the study protocol (24).

## Discussion

The ‘High schools High on life’ intervention is designed to provide important insights into effective strategies to reduce excessive alcohol consumption among adolescents. The intervention was systematically designed using the planning steps of the IM protocol (26), the behavior change wheel (56) and guided by theory, evidence, and empirical findings. The development process resulted in a multi-component intervention with the following intervention elements: a school environmental component targeting school alcohol policies and norms, student components addressing students' social norms around alcohol use and parental components addressing parent-child communication about alcohol use.

Not all steps of IM were followed rigidly. However, we found it very useful as a planning tool in combination with the behavior change wheel and the behavior change techniques, as it provided a systematic approach to the development process and as it forced us to be explicit about our choices throughout the planning process. According to the IM protocol, the most important and modifiable determinants should be chosen for intervention (26). We identified the most important modifiable determinants within the high school environment for intervention including environmental and social factors. However, our design may have some limitations. In the context and capacity assessment schools were selected based on experience with interventions related to alcohol. We only interviewed a small number of school principals and students which may have drawn our attention to specific challenges and barriers in some contexts that may not be generalizable to all Danish high schools. However, in our testing of intervention ideas and components we chose to include high schools from both city and rural areas as well as different regions of the country to capture the heterogeneity of high schools. We interviewed both principals and students at schools that already had been working with alcohol prevention interventions and principals and students at schools that had not, to identify different possible barriers to the intervention and make our results as representative as possible.

The school is only one of the arenas of young people's everyday life - there are many arenas where young people meet and engage in heavy drinking that are not targeted by the intervention which may dilute the potential intervention effects. One specific drinking occasion is pre-partying before high school parties. This is often where the largest number of alcoholic drinks is consumed in connection with high school parties. We were unable to design feasible components targeting pre-parties, as interviews with parents showed that they are seldom present at pre-parties and generally don't feel responsible for pre-parties. Peer groups established in high schools do not cease when the students leave school at the end of the school day, go on weekend outings or holidays but continue outside the school setting. As such, it can be assumed that norms created in the school setting will be transferred to social settings outside the school.

We also identified several important, but non-modifiable determinants of excessive drinking in adolescence e.g., societal and community factors that effects alcohol availability and drinking norms, including the low purchasing age (16 years) for alcohol in Denmark, low enforcement of legal purchasing age, liberal alcohol norms, and cheap prices for alcohol. These are all important determinants but were beyond our ability to change.

In addition to targeting the most important and modifiable determinants of adolescent and young adults drinking, we found there is a third important factor to consider when selecting intervention components: the implications of selection and recruitment of schools to take part in the study. The intervention design had to balance intensity of intervention and easy implementation to recruit schools and ensure components would be appealing and feasible to implement for intervention schools. This affected the content of the intervention, for example a total ban of alcohol at high schools was not something schools were interested in implementing. Beside the primary intervention outcome of reduction in binge drinking, the intervention is also expected to promote inclusion of students who drink moderately or do not want to drink, and to promote a positive school culture with social events not focusing on alcohol. The additional focus on promotion on more positive outcomes, were found to be more appealing to school management than the focus of reduction of alcohol consumption alone. The intervention tried to reflect high schools' values of educating students to be responsible and knowledgeable and tried to give schools a way of promoting themselves as responsible.

The transparency of the developmental process and theoretical, empirical, and practical/contextual foundation of the ‘High schools High on life’ intervention (Table A1) enables future intervention studies to build on our findings and accumulate knowledge on how to decrease young peoples' excessive drinking. In combination with the randomized controlled trial design, the comprehensive mixed methods process evaluation of our intervention will illustrate the amount of implementation support needed from the project group to implement the intervention and possibly affect the number of binge drinking episodes.

We found the collaboration between research and practice very fruitful as the DCS had special communication and pedagogical competences and experience with alcohol prevention in the target group that supplemented the scientific competences in the research group. The ‘High schools High on life’ intervention was designed to be sustainable and up scalable to a national recommendation. The DCS owns the materials and can carry forward implementation on a national level.

## Conclusion

The ‘High schools High on life’ intervention was designed to reduce excessive drinking among high school students. It was designed systematically inspired by the planning steps of the IM protocol (26), the behavior change wheel (56) and was guided by theory, evidence, and empirical findings. The development process resulted in a multi-component intervention with the following intervention elements: a school environmental component targeting school alcohol policies and norms, student components addressing students' social norms around alcohol, and parental components addressing parent-child communication around alcohol. The transparent description of all planning steps will enable future alcohol prevention programs to build on our intervention and evaluation design and accumulate knowledge to help decrease excessive drinking among young people worldwide.

## Data Availability Statement

The datasets generated and analyzed during the current study are not publicly available due to sensitivity of the data but are available from the corresponding author on reasonable request.

## Ethics Statement

In Denmark, behavioral health promotion interventions are generally not required to notify for ethic approval by the Scientific Ethics Committees (75). The Scientific Ethics Committees for the Capital Region of Denmark has declared that the trial is not subject to notification (jnr. 19021957). The study is registered at the Research an Innovation Office at University of Southern Denmark (ref: 10.314) allowing collection of personal data. When inviting the high schools to participate, school principals received written information about the study. For all data collection methods, responders were informed about the aim of the study, that participation was voluntary, that their information would be used for research purposes only and would be treated confidentially. In the written introduction to the electronic questionnaires, responders were asked to agree that they have received information about the study and have agreed to the use of their data for research and consent to participate. Participants could skip questions they did not wish to answer. For the qualitative data collection consent to participate was verbal. According to Danish law children can give consent based on their maturity and children of the aged of 13 years and above can give consent for use of their personal data.

## Author Contributions

MG and JT had the original idea for the study and applied for funding. VP wrote the first draft of the manuscript with SH's assistance. VP and SH coordinated the development of the intervention design with RK providing advice on the use of IM and BCTs. LP and PD led the production of intervention materials. VP and SH conduced the interviews with students and high school staff. VP and RK coded the intervention components according to intervention function, policy categories, and BCTs. LT, JT, MG, and RK advised the intervention design. All authors read, revised, and approved the final manuscript.

## Conflict of Interest

The Danish Cancer Society developed intervention materials for the ‘High schools High on life’ project based on an ongoing campaign. The Danish Cancer Society will have no influence on the evaluation study design, data analysis or interpretation of data. The remaining authors declare that the research was conducted in the absence of any commercial or financial relationships that could be construed as a potential conflict of interest.

## Abbreviations

BCTs: Behavior Change Techniques
CIR: Centre for intervention Research
DCS: Danish Cancer Society
IM: Intervention Mapping.

## Appendix

**Table A1:** Template for Intervention Description and Replication.

**Brief name**	**POLICY CATEGORY and Intervention functions**	**BCT**	**Who is the target group?**	**Why?**	**What?**	**Who provided?**	**How?**	**Where?**	**When and How Much?**	**Tailoring?**
**School Environmental Component**										
Alcohol policy	REGULATION Restrictions Environmental restructuring COMMUNICATION/ MARKETING	12.2 Restructuring the social environment 12.3 Restructuring the physical environment	School management	To limit availability of alcohol, communicate a clear attitude to alcohol on the school, and to ensure enforcement of policy The checklist was based on evidence from national alcohol policies (58, 59) which have shown effect previously	An alcohol policy checklist with mandatory points and recommendations	The checklist was developed by DCS^1^ and CIR^2^. The project coordinator will be responsible for the implementation	Project coordinators receive the checklist, discuss policy and enforcement with relevant personnel, revise alcohol policy by including missing mandatory elements from the alcohol policy checklist, and inform parents, students and personnel	The alcohol policy should be enforced and communicated to parents, personnel and students	The policy checklist must be returned to CIR prior to school start August 2019. Parents and students must be informed about the policy in the beginning of the school year	Each school can decide if they also want to introduce the optional elements of the checklist Other materials: -An implementation guide on how to work with the policy and improve enforcement. -Guidelines for the development of a strategy for referral to treatment of students that may have a substance abuse. -Guidelines on tasks and responsibility for personnel present at social school events where alcohol is sold
Web-based Education for Students in Party- and Introduction Committees	COMMUNICATION/MARKETING Education Persuasion Training Modelling REGULATION Environmental restructuring	4.1 Instructions on how to perform the behavior 5.3 Information about social and environmental consequences 6.3 Information about others' approval 12.2 Restructuring the social environment 13.1 Identification of self as role model	Students in Party- and Introduction Committees	To make the members aware of peer pressure on alcohol and their position as role models, and enable them to arrange socially inclusive events with no promotion of excessive drinking Based on evidence of effectiveness of peer-led interventions (60, 61)	Educational material: Quiz with information of legislation on alcohol promotion, peer pressure on alcohol, social responsibility and recognizable dilemmas for discussion Students in Party Committees further receive an innovative guided workshop on how to plan fun events and party activities not focusing on drinking	The material was developed by DCS The introduction and party committee coordinators should arrange time and place of the committee(s) to complete the education, provide post its, pens and print materials	The respective coordinator of the students in Party- and Introduction Committees will introduce the intervention to the committee and ensure that they complete the web-based education	Two lesson (90min) at school	Students in Introduction Committees: Once before planning the introduction week Students in Party Committees: Once in the beginning of the school year Lessons learned should guide the committees work throughout the year	The students can decide which activities they find appealing and how they should be organized. The coordinator decides which of the following material they will involve in the education Materials: -Dilemma-cards -Contract of responsible conduct and certificate for being member of a responsible student committee -Power point template with information about the project and the committee's task and responsibilities -Fact sheet on youth and alcohol Students in Introduction Committees: -Catalog of ideas for social activities and name-games -Suggestion for schedule for introduction days. -Letter to new 1^st^ year students- template, guidance and example Students in Party Committees: -Templates for party invitations
**School Educational Component**										
Pocket Movie Campaign/ Workshop	COMMUNICATION/ MARKETING Education Persuasion	5.3 Information about social and environmental consequences. 5.4 Information about emotional consequences	1^st^ year students	The aim was to make 1^st^ year students reflect on their alcohol use when it is fun, and to promote drinking less as the ideal behavior. Based on Induced compliance theory (64)	Pocket movie campaign competition	A full day workshop will be delivered by Lommefilm A/S and DCS. The high school should provide one teacher per class to help facilitating the process	Lommefilm A/S will facilitate a one-day workshop for all 1^st^ year students on how to make movies using their smartphones and knowledge on short- and long-term consequences of heavy drinking. In groups of 4 students create a campaign movie with the message “Drink less- experience more”. The best movie at the school is selected and will participate in a national competition	At the school one school day	One day during the first school year (October – December)	No
Social Norms Campaign	COMMUNICATION/ MARKETING Education Persuasion Modelling	5.3 Information about social and environmental consequences. 5.4 Information about emotional consequences 6.2 Social comparison 6.3 Information about others' approval	1^st^ year students (primary)	To correct misinformation of peers' attitude and behavior in relation to alcohol. Guided by the Social Norms Approach	Video campaign where 3^rd^ year students advise 1^st^ year students not to drink too much. Posters (digital and print) with data on behavior and attitude to alcohol among students at one's high school	DCS created the video and posters. CIR provided the data for the posters Each school should make materials visible in physical surroundings and online platforms	The materials will be sent to the school and distributed on their online platforms and/or physical surroundings	Campaign movie targeted to new high school students at intervention schools on Facebook. Posters at the school	Throughout the year	Targeted each school Other materials: -Power point template with information about the school's attitude to alcohol and the alcohol policy to be presented for all students
Morning Party Event	COMMUNICATION/ MARKETING Training Persuasion	8.1 Behavioral rehearsal/practice. 8.2 Behavior Substitution 8.4 Habit reversal 12.2 Restructuring the social environment	1^st^ year students (primary)	To give students an experience of parting without drinking that can be transferred into practice at other parties	Morning party event	DCS created the concept of the morning party and supported the event	DCS will contact the schools and suggest the schools to host a morning party. DCS will support the planning and organizing of the event with 10.000 DKK	At the school	One hour once in the middle of the school year	Voluntary element
**Parent Component**										
Information Meeting	COMMUNICATION/ MARKETING Education	4.2 Information about antecedents	Parents of 1^st^ year students	To give parents information about the school policy and inform them of their responsibility in relation to e.g. preparties	Power point template with information about the school's attitude to alcohol and the alcohol policy	DSC developed the power point template. The school management will organize the meeting and present the alcohol policy	Face to face	At the school	Once in the beginning of the school year	Each school could modify presentation to reflect the school policy. Other materials: -Power point for the parent meeting
Information Folder	COMMUNICATION/ MARKETING Education Persuasion	4.1 Instructions on how to perform the behavior. 4.2 Information about antecedent 6.3 Information about others approval	Parents of 1^st^ year students	To make parents aware of their responsibility and influence on their child's drinking and encourage them to talk to their child about alcohol and make agreements on their child's consumption	Information folder	DCS	Information folder handed out to parents at the high school	At the parent meeting at the school	In the beginning of the first school year	No.
Information Website	COMMUNICATION/ MARKETING Education Persuasion	4.1 Instructions on how to perform the behavior. 4.2 Information about antecedents 5.1 Information about health consequences 5.3 Information about social and environmental consequences 6.3 Information about others approval	Parents of 1^st^ year students	Give parents information on alcohol consumption among adolescents, their impact on their child's consumption and negative consequences of alcohol consumption. Provide the parents skills to discuss alcohol their child	Information website, including quiz, games with skill training on deciding rules, movies with psychologist giving advises on dialog and agreements on alcohol with children, movie with students talking about acceptance of parental involvement in their alcohol use, and written information	DCS High school makes parents aware of the website, and the information folder refers to the website	Website	Online	Not specified	No.

1 *DSC = The Danish Cancer Society*;

2 *CIR = The Centre of Intervention Research, National Institute of Public Health, Southern Denmark University*.
